# Case Series of SMARCA4-Deficient Undifferentiated Esophageal Carcinoma

**DOI:** 10.7759/cureus.30874

**Published:** 2022-10-30

**Authors:** Omar T Ahmed, Ga Hie Nam, Yuan Shui, Jaimy Villavicencio, Haleh Vaziri

**Affiliations:** 1 Department of Internal Medicine, University of Connecticut, Farmington, USA; 2 Department of Pathology and Laboratory Medicine, University of Connecticut, Farmington, USA; 3 Department of Gastroenterology and Hepatology, University of Connecticut, Farmington, USA

**Keywords:** malignancy, tumor, brg1, carcinoma, esophageal, undifferentiated, deficient, smarca4

## Abstract

Undifferentiated esophageal carcinomas (UEC) are rare, with aggressive behavior and a dismal prognosis. An extremely rare subset is the SMARCA4-deficient UEC, which has only been reported in 14 cases to date. We present two cases of male patients (39- and 64-year-old) with SMARCA4-deficient UEC. Both patients had evidence of metastatic disease on presentation, progressed rapidly, and passed away within three months from the presentation. We aim to raise awareness of this underreported disease and contribute to the exploration of the possible underlying pathology and risk factors.

## Introduction

This article was presented as a poster abstract at the 2022 ACG Annual Meeting on October 24, 2022.

Esophageal cancers rank as the eighth most common malignancy, with adenocarcinoma being the most common in the United States [1]. Undifferentiated esophageal carcinoma (UEC) is a rare histologic variant that was recently recognized as a separate entity by the World Health Organization. It is associated with aggressive behavior, with a one-year survival rate of approximately 25%. Its prevalence ranges from 0.2%-4.0% due to the lack of clear diagnostic criteria [2]. The SMARCA4 gene encodes the BRG1 protein, which has a tumor suppressor role. UEC with a SMARCA4 gene deficiency is an extremely rare disease, with only 14 cases previously reported. We present two new cases with SMARCA4-deficient UEC.

## Case presentation

Case 1

**Figure 1:**
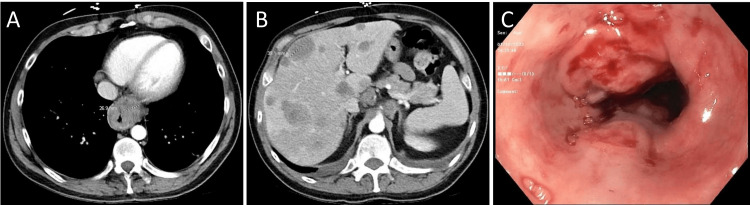
Case 1 images A: CT showing an esophageal heterogeneous mass that is 26.9 mm thick in the distal third of the esophagus. B: CT showing innumerable solid hypodense hepatic masses (largest measuring 39.1 mm) with associated upper abdominal lymphadenopathy. C: EGD showing a large, non-obstructing, circumferential, ulcerating mass in the lower third of the esophagus. EGD: esophagogastroduodenoscopy

A previously healthy 39-year-old Caucasian male presented with fevers, nausea, right upper quadrant (RUQ) abdominal pain, and a 15 lb weight loss over the preceding month. No significant social history was reported. He reported esophageal cancer in his maternal grandfather in his seventh decade of life. On presentation, he was febrile and tachycardic, with mild RUQ tenderness and hepatomegaly. Laboratory workup was unremarkable. Abdominal computed tomography (CT) showed a distal esophageal mass with hepatic metastasis (Figures 1A, 1B). Esophagogastroduodenoscopy (EGD) revealed a large, ulcerating distal esophageal mass (Figure 1C), which was biopsied. The patient developed progressively worsening symptoms and eventually passed away 1.5 months after his initial presentation.

Case 2

**Figure 2:**
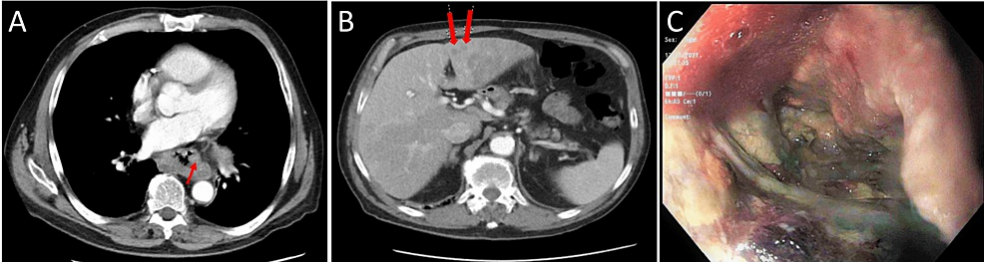
Case 2 images A: CT abdomen showing irregular thickening of the distal esophagus and neoplastic infiltration and necrosis along the lateral walls of the esophagus (red arrow). B: CT showing multiple hepatic metastases (red arrows); the largest was 18.4 mm (not shown). C: EGD showing a fungating circumferential, necrotic, distal esophageal ulcer with stigmata of recent bleeding. EGD: esophagogastroduodenoscopy

A 64-year-old Caucasian man, with a history of hiatal hernia and reflux disease, presented with intermittent, burning chest pain. He reported smoking tobacco and alcohol use. Family history was non-contributory. Initial exam and laboratory workup were unremarkable, so the patient was started on a proton pump inhibitor (PPI) and discharged home. Two months after the index presentation, he represented with persistent symptoms despite being on PPI therapy, and this time, an abdominal CT showed infiltrating distal esophageal mass with hepatic metastases (Figures 2A, 2B). EGD revealed a circumferential, necrotic, distal esophageal ulcer (Figure 2C). Due to his significant metastatic liver disease, the patient did not qualify for chemotherapy and eventually passed away three months after the initial presentation.

Pathology findings

**Figure 3:**
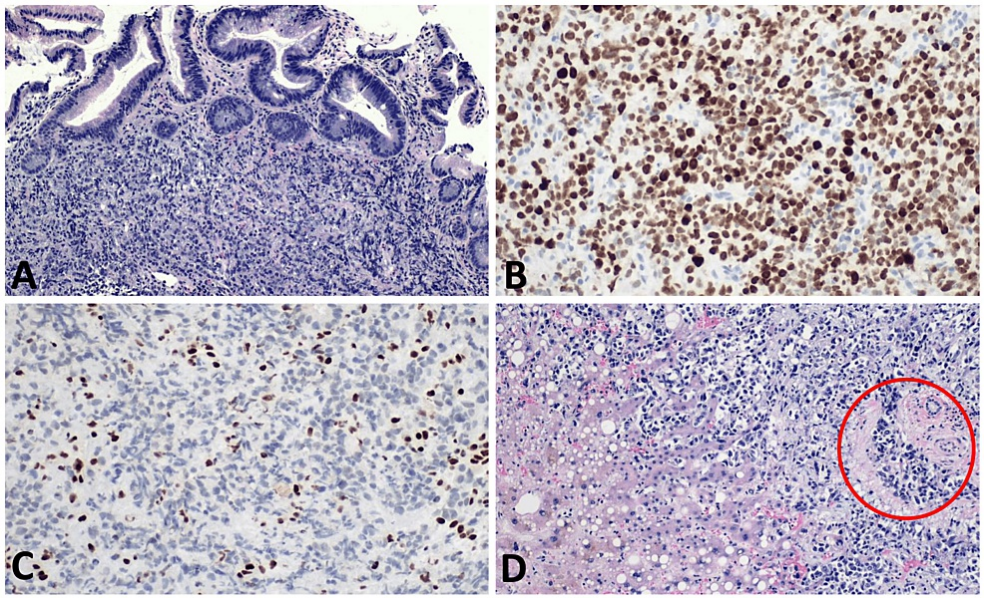
Pathology findings in both cases A: Both tumor biopsies showed undifferentiated tumor cells consisting of epithelioid cells arranged in small clusters with overlying intestinal metaplasia (seen as columnar cells with goblet cells on top of the tumor cells). B: Tumor cells strongly positive for SALL4, only in Case 1. C: Loss of SMARCA4/BRG1 within the tumor cells while retained within inflammatory cells (internal control). D: Diffuse infiltration of liver parenchyma with tumor cells, predominantly within the hepatic sinusoids and portal triads (red circle) in Case 2.

The esophageal tumor biopsy in both cases showed undifferentiated malignant tumor cells in small clusters to solid sheets with no glandular or squamous differentiation (Figure 3A). The overlying mucosa showed intestinal metaplasia consistent with Barrett’s esophagus (BE). Focal staining with epithelial markers suggested an epithelial origin. Tumor cells were strongly positive for Spalt Like Transcription Factor 4 (SALL4) only in Case 1 (Figure 3B). However other germ cell markers, neuroendocrine, squamous, melanocytic, and vascular markers were all negative. Loss of expression of SMARCA4/BRG1 within tumor cells was noted (Figure 3C) while stains for SMARCB1/INI1 were positive. These findings are most consistent with SMARCA4-deficient UEC.

**Figure 4:**
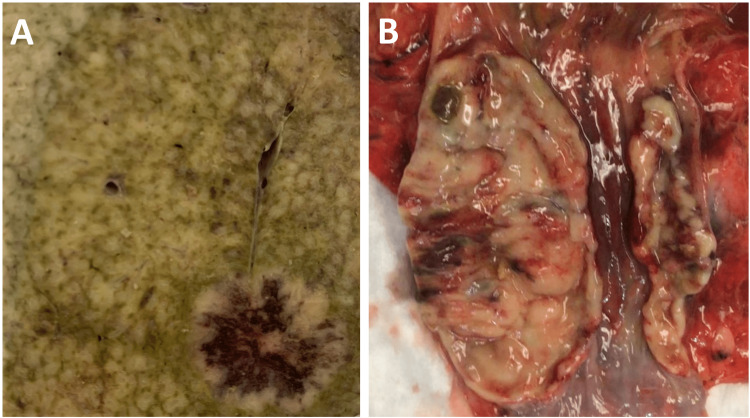
Postpartum pathology of Case 2 A: 16 mm liver metastasis (lower right) in a background of diffuse yellow-mottled appearance, consistent with an extensive tumor infiltration within the hepatic sinusoids. B: Near circumferential, 10 cm, necrotic, distal esophageal mass extending through the lateral esophageal walls.

Postmortem pathology of Case 2 showed a massively enlarged liver (4500 g) with extensive metastatic infiltration in all the parenchyma (Figure 3D, Figure 4A), and a near circumferential, 10 cm distal esophageal mass extending through the lateral esophageal walls (Figure 4B).

## Discussion

The SMARCA4 gene encodes the BRG1, a chromatin remodeling protein, which increases chromatin accessibility allowing for double-strand damage repair [3], hence thought to have a tumor suppressor role. The loss of the SMARCA4 gene has been associated with undifferentiated highly aggressive tumors in different organs, including the GI tract.

UEC has been only reported as part of epidemiologic studies or case reports [4-10] and was recently recognized as a distinct entity, however, its clinicopathological features were undefined [2]. SMARCA4-deficient UEC is an even more underreported diagnosis with a total of 16 cases reported to date (summarized in Table 1).

**Table 1:** All the reported cases of SMARCA4-deficient undifferentiated esophageal carcinoma * Reported by Horton et al. [6]; ^ Reported by Kilic et al. [7]; + Reported by Tessier-Cloutier et al. [10]; ^^ Our cases. E: esophageal (not otherwise specified); DE: distal esophageal; ME: mid-esophageal; GEJ: gastroesophageal junction; M: male; F: female; NA: not available

Number	Age at diagnosis	Gender	Location	Presence of BE	SMARCA4/BRG1	SMARCB1/INI
1*	63	M	GEJ	No	Absent	Retained
2*	72	M	E	Yes	Absent	Retained
3*	77	F	ME	Yes	Absent	Retained
4*	72	F	ME	Yes	Absent	Retained
5*	70	M	DE	Yes	Absent	NA
6*	64	M	E	Yes	Absent	NA
7*	68	F	GEJ	Yes	Absent	NA
8*	76	M	DE	NA	Absent	Retained
9*	63	M	DE	Yes	Absent	NA
10*	79	F	DE	Yes	Absent	Retained
11*	77	M	E	No	Absent	NA
12*	73	M	DE	NA	Absent	Retained
13^^^	70	M	GEJ	NA	Absent	Retained
14^+^	79	M	GEJ	NA	Absent	Retained
15^^^^	64	M	DE	Yes	Absent	Retained
16^^^^	39	M	DE	Yes	Absent	Retained

In 2021, Horton et al. reported the first case series of 12 patients with SMARCA4-deficient UEC [6]. The disease was predominantly seen in elderly men, consistent with other reports on UEC or SMARCA4-deficient neoplasms [7-10]. Our 64-year-old male patient fits the commonly reported demographics while our 39-year-old male patient is the youngest patient reported to present with SMARCA4-deficient UEC.

Histologically, BE was observed in the background of tumor cells in our cases. This finding was also observed in eight of 12 SMARCA4-deficient UEC cases reported by Horton et al., among which three cases had high-grade dysplasia, and one showed progression from BE to dysplasia, to adenocarcinoma, and then to SMARCA4-deficient UEC [6]. Similar findings were described in a case series by Singhi et al, who reported 16 cases of UEC, 12 of which had BE in the background. In addition, five cases had dysplasia within BE, and three cases had focal adenocarcinoma at the peripheries. The SMARCA status was not reported in this series [8]. The presence of BE and the distal esophageal location in these tumors suggests the possibility of UEC arising from progressive de-differentiation of BE with or without developing adenocarcinoma as an intermediate step.

Unfortunately, there is almost no data on individual risk factors for UEC. Even less is known about hereditary predisposition. The second patient had risk factors for esophageal carcinoma while the first patient had a positive family history, which highlights the possibility of a germline mutation. Duan et al. reported a patient with SMARCA4-deficient undifferentiated colonic carcinoma due to a germline SMARCA4 gene mutation, who had a second-degree relative with colon cancer at age 50 [11].

There is no effective treatment for SMARCA4-deficient UEC. Case reports have demonstrated successful treatment with immune checkpoint blockades (ICBs) in other SMARCA4-deficient tumors with high tumor mutation burden [12,13] and with overexpression of programmed cell death ligand 1 (PDL-1) proteins [14]. Other suggested therapeutic options are CDK4/6 inhibitors [15] and targeting SALL4 [16,17]. SALL4 was strongly expressed in Case 1, which suggests a role in tumor pathogenesis and hence a potential therapeutic target.

## Conclusions

Like most undifferentiated tumors, UEC is known to have a grim prognosis, allowing very limited time for therapeutic trials. The rapid progression and aggressive nature of the disease, along with the limited availability and use of SMARCA4 immunostaining could explain the underreporting of this disease. SMARCA4 immunostains should be considered for prognostication purposes and possibly treatment selection. An effective treatment is yet to be discovered but emerging data on potential therapeutic targets, such as PD-L1, CDK4/6, and SALL4, is encouraging. Lastly, the possibility of germline mutations affecting younger patients, as well as its association with BE as a possible precursor lesion, should be considered and further studies are needed to further investigate this.
